# Significant Publications on Infectious Diseases Pharmacotherapy in 2024

**DOI:** 10.1177/08971900251408307

**Published:** 2025-12-11

**Authors:** Lauren Lee, Yao-Hsuan Huang, Hiba Al Shaikhli, Parijat Billah, Nhi Nguyen, Jamie Thomas, Ahmed Zaki, Kevin Lin

**Affiliations:** 1 Department of Pharmacy, 12338 The University of Texas Medical Branch, Galveston, TX, USA; 2 Department of Pharmacy, 23534 Houston Methodist Hospital, Houston, TX, USA; 3 Department of Pharmacy, 173819 Memorial Hermann Sugar Land, Sugar Land, TX, USA; 4 Department of Pharmacy, 91304 Harris Health Ben Taub Hospital, Houston, TX, USA; 5 Department of Pharmacy, 23469 Memorial Hermann Southwest, Houston, TX, USA; 6 Department of Pharmacy, 23467 Memorial Hermann Greater Heights, Houston, TX, USA; 7 Division of Pharmacy, 4002 The University of Texas MD Anderson Cancer Center, Houston, TX, USA

**Keywords:** infectious diseases, human immunodeficiency virus, pharmacotherapy, antimicrobial stewardship, anti-infective

## Abstract

**Purpose:** To provide a summarization of the most significant infectious diseases (ID) pharmacotherapy articles published in peer-reviewed literature in 2024. **Summary:** Members of the Houston Infectious Diseases Network (HIDN) nominated notable articles providing significant contributions to ID pharmacotherapy in 2024. Article nominations included those pertaining to general ID and human immunodeficiency virus/acquired immunodeficiency syndrome (HIV/AIDS) pharmacotherapy in 2024. To aid selection of the most significant articles in 2024, a survey was created and distributed to members of the Society of Infectious Diseases Pharmacists (SIDP). Out of the 21 total articles nominated by HIDN/SIDP members, 19 pertained to general ID pharmacotherapy, and 2 had pertained to HIV/AIDS pharmacotherapy. Of the SIDP members who participated in the survey, 185 voted for the top 10 general ID pharmacotherapy articles and 46 votes were recorded for the top HIV/AIDS article. The most notable publications are summarized. **Conclusion:** This review provides a summary of the most recently published ID literature with aims to update clinicians on the current potential practice changing ID pharmacotherapy publications from 2024.

## Introduction

As the infectious diseases (ID) community persists to navigate a post-pandemic world, there remains continued progress in ID-related publications given the everchanging landscape of infectious pathogens and treatment considerations. The Houston Infectious Diseases Network (HIDN), consisting of multidisciplinary infectious diseases providers from over 15 different institutions in and around the Texas Medical Center located in Houston, Texas, promotes research and antimicrobial stewardship through publication and dissemination of current literature and advancements in the ID realm. Since 2007, HIDN has complied a yearly review of the top 10 most significant publications in ID pharmacotherapy with the goals of providing clinicians with an overview of the advances in ID research in the past year^1-17^ Members of HIDN include ID physicians, pharmacists, microbiologists, and others that meet regularly to advance the practices of ID through collaborative research, education, and implementation of best practices and new stewardship strategies.

## Article Selection Process

Members of HIDN were requested to nominate the most influential peer-reviewed ID pharmacotherapy articles, excluding their own research, published between January 1 and December 31, 2024. Article nominations were requested via email over a 4-week period with reminders during week 3 and 4. Upon conclusion of the nominations, 17 total articles were nominated, 15 general ID and 2 HIV/AIDS publications, for submission to members of the Society of Infectious Diseases Pharmacists (SIDP) for voting to rank the top 10 general ID and top HIV/AIDS publication felt to contribute significantly to ID pharmacotherapy. SIDP, an organization of over 2000 infectious diseases pharmacists, sent an electronic survey to its members over a 2-week period. SIDP members were able to vote for up to 10 general ID articles and only 1 HIV/AIDS article. They were also afforded the opportunity to vote for articles not included in the initial list of nominations, with each selection counting as a single vote. Members could abstain from voting in either category of article for any reason. One hundred and eighty-five SIDP members in the United States voted for the top 10 general ID publications and 46 voted for the top HIV/AIDS publication. At the conclusion of the survey, an additional 4 general ID articles were nominated by SIDP for a grand total of 21 articles, 19 general ID and 2 HIV/AIDS publications. The final ranking of publications was determined by total votes per survey response. The rankings of the ID pharmacotherapy and HIV/AIDS publications are listed in Tables 1 and 2, respectively. The top 10 general ID and top 1 HIV/AIDS papers are summarized here in alphabetical order of the first author.^18-32,37^

**Table 1.** Results of SIDP Member Ranking of Significant Publications on Infectious Diseases Pharmacotherapy in 2024

Rank	Reference	Article	Number (%) individuals ranking article in top 10 (n = 185)
1	18	Daneman N et al. Antibiotic treatment for 7 vs 14 days in patients with bloodstream infections	95 (51%)
2	19	Kaasch AJ et al. Efficacy and safety of an early oral switch in low-risk *Staphylococcus aureus* bloodstream infection (SABATO): an international, open-label, parallel-group, randomised, controlled, non-inferiority trial	93 (50%)
3	20	López-Cortés LE et al. Efficacy and safety of a structured de-escalation from antipseudomonal β-lactams in bloodstream infections due to Enterobacterales (SIMPLIFY): an open-label, multicentre, randomised trial	76 (41%)
4	21	Tamma PD et al. Infectious diseases society of America 2024 guidance on the treatment of antimicrobial-resistant gram-negative infections	75 (41%)
5	22	Tingsgård S et al. Early switch from IV to PO antibiotics for patients with uncomplicated gram-negative bacteremia	75 (41%)
6	23	Gohil SK et al. Stewardship prompts to improve antibiotic selection for pneumonia: the INSPIRE randomized clinical trial	74 (40%)
7	24	Dulhunty JM et al. Continuous vs intermittent β-lactam antibiotic infusions in critically ill patients with sepsis: The BLING III randomized clinical trial	73 (39%)
8	25	Gohil SK et al. Stewardship prompts to improve antibiotic selection for urinary tract infection: The INSPIRE randomized clinical trial	72 (39%)
9	26	Karaba SM et al. Extended-infusion β-lactam therapy, mortality, and subsequent antibiotic resistance among hospitalized adults with gram-negative bloodstream infections	59 (32%)
10	27	Ranganath N et al. Short vs prolonged duration of therapy for *Pseudomonas aeruginosa* bacteraemia: a systematic review and meta-analysis	55 (30%)
11	28	Kam KW et al. Epidemiology and outcomes of antibiotic de-escalation in patients with suspected sepsis in US hospitals	53 (55%)
12	29	Mo Y et al. Individualised, short-course antibiotic treatment vs usual long-course treatment for ventilator-associated pneumonia (REGARD-VAP): a multicentre, individually randomised, open-label, non-inferiority trial	50 (27%)
13	30	Nussbaum EZ et al. Oral antibiotics for treatment of gram-negative bacteremia in solid organ transplant recipients: a propensity score weighted retrospective observational study	42 (23%)
14	31	Dark P et al. Biomarker-guided antibiotic duration for hospitalized patients with suspected sepsis: the ADAPT-sepsis randomized clinical trial	40 (22%)
15	32	Laporte-Amargos J et al. Efficacy of extended infusion of β-lactam antibiotics for the treatment of febrile neutropenia in haematologic patients (BEATLE): a randomized, multicentre, open-label, superiority clinical trial	33 (18%)
16	33	Shields RK et al. Effectiveness of ceftazidime-avibactam vs ceftolozane-tazobactam for multidrug-resistant *Pseudomonas aeruginosa* infections in the USA (CACTUS): a multicentre, retrospective, observational study	2 (1%)
17	34	Teshome BF et al. Preventing new gram-negative resistance through beta-lactam de-escalation in hospitalized patients	1 (1%)
18	35	Montini et al. Short oral antibiotic therapy for pediatric febrile urinary tract infections: a randomized trial	1 (1%)
19	36	Eubank TA et al. Reduced vancomycin susceptibility in *Clostridioides difficile* is associated with lower rates of initial cure and sustaind clinical response	1 (1%)

**Table 2.** Results of SIDP Member Ranking of Significant Publications on HIV/AIDS Pharmacotherapy in 2024

Rank	Reference	Article	Number (%) individuals ranking article in top 10 (n = 46)
1	37	Bekker LG et al. Twice-yearly lenacapavir or daily F/TAF for HIV prevention in cisgender women	36 (78%)
2	38	Christopoulos KA et al. Learning from the first: A qualitative study of the psychosocial benefits and treatment burdens of long-acting cabotegravir/rilpivirine among early adopters in three U.S. clinics	10 (22%)

### Bekker LG et al. Twice-Yearly Lenacapavir or Daily F/TAF for HIV Prevention in Cisgender Women

Multiple pre-exposure prophylaxis (PrEP) options are currently available to prevent HIV infection. Among them, emtricitabine-tenofovir disoproxil fumarate (FTC-TDF) and emtricitabine-tenofovir alafenamide (FTC-TAF) are two effective oral options.^39-41^ Despite proven efficacy with the use of PrEP, cisgender women, who account for nearly half of new HIV infections globally each year, face adherence challenges and remain a vulnerable population.^42-44^ Lenacapavir, a long-acting HIV-1 capsid inhibitor, was approved in December 2022 by the Food and Drug Administration (FDA) for multidrug-resistant HIV in heavily pre-treated adults and is now under FDA review for use as PrEP.^45,46^

PURPOSE 1 was a phase 3, multicenter, double-blind, randomized, controlled trial conducted in South Africa and Uganda to assess the efficacy and safety of twice-yearly subcutaneous lenacapavir and daily oral FTC-TAF for HIV prevention, compared to FTC-TDF and background HIV incidence. The study screened sexually active women aged 16 to 25 without prior PrEP use, no HIV testing in the past three months, and unknown HIV status. Eligible participants were included in a cross-sectional background HIV incidence group and underwent HIV testing. HIV-negative participants were randomized to receive subcutaneous lenacapavir 927 mg every 26 weeks, daily oral FTC-TAF 200-25 mg, or daily oral FTC-TDF 200-300 mg (2:2:1), with matched placebo tablets or injections for blinding. The primary efficacy endpoint was HIV incidence. Secondary endpoints included adverse events, clinical laboratory abnormalities, and adherence.

A total of 8094 participants were included in the cross-sectional background HIV group, and 5345 underwent randomization. Among 5338 participants who were initially HIV-negative (median age [range], 21 [16-26]), 2134 received lenacapavir, 2136 received FTC-TAF, and 1068 received FTC-TDF. Baseline demographics were similar across all treatment groups and background HIV group. No HIV infections were observed in the lenacapavir group (0 per 100 person-years; 95% CI 0.00 to 0.19), 39 in the FTC-TAF group (2.02 per 100 person-years; 95% CI 1.44 to 2.76), and 16 in the FTC-TDF group (1.69 per 100 person-years; 95% CI 0.97 to 2.74). Background HIV incidence was 2.41 per 100 person-years (95% CI 1.82 to 3.19). HIV incidence in lenacapavir group was significantly lower than both background HIV incidence group (incidence rate ratio, 0.00; *P* < 0.001) and the FTC-TDF group (incidence rate ratio, 0.00; *P* < 0.001). HIV incidence did not significantly differ between FTC-TAF and background HIV incidence group (*P* = 0.21) or between FTC-TAF and FTC-TDF groups (95% CI 0.67 to 2.14). Plasma drug concentration measurements indicated low adherence in both FTC-TAF and FTC-TDF groups. Most adverse events and laboratory abnormalities were similar across all groups, except more injection site reactions (68.8% vs 35.3% vs 33.9%) and less nausea (6.7% vs 10.9% vs 13.3%) and vomiting (5.8% vs 11.0% vs 10.0%) were observed in the lenacapavir group.

Limitations include generalizability to other regions, as patients included were from South Africa and Uganda. The study also excluded older women, and those with comorbidities that could impact drug metabolism. Despite these limitations, this study showed that twice-yearly subcutaneous lenacapavir is an effective and well-tolerated PrEP option, offering a potential solution to improve adherence and HIV prevention in cisgender women.

### Daneman N et al. Antibiotic Treatment for 7 vs 14 Days in Patients With Bloodstream Infections

There are currently few evidence-based practice guidelines available to aid in broadly determining treatment duration in patients with bloodstream infections. These specific guidelines have a narrow scope in treating either particular pathogens (eg, *Staphylococcus aureus*, *Candida* spp.) or indications (eg, catheter-related bloodstream infections).^47-49^ Various smaller clinical trials that have been recently conducted have demonstrated similar clinical and microbiological outcomes in patients receiving shorter courses of antibiotic therapy (ie, 7 days) vs longer courses (ie, 14 days).^50-53^ Although, these trials primarily focused on treatment outcomes for uncomplicated gram-negative bacteremia and did not encompass a wider group of pathogens.

The BALANCE trial was a multicenter, open-label, randomized controlled, noninferiority trial across 74 hospitals in seven countries from October 2014 to May 2023 to assess 7 days of antibiotic therapy compared to 14 days in hospitalized patients with bacteriemia. Eligible patients were admitted at the time of positive blood culture with a pathogenic bacterium. Patients were excluded if severely immunocompromised (ie, neutropenia or immunosuppressive therapy after solid organ or hematopoietic stem-cell transplant), had prosthetic heart valve or endovascular grafts, *S*. *aureus* or *Staphylococcus lugdunensis* bacteria, fungemia, required prolonged treatment (ie, endocarditis, osteomyelitis, etc.), or had a positive culture with a common skin contaminant (ie, coagulase-negative *Staphylococci*). The primary outcome was death at 90 days after bacteremia diagnosis.

Over 13 597 patients met eligibility criteria for the trial with 3631 (26.7%) enrolled. Baseline characteristics, pathogens, and treatments were similar between groups. Most infections were classified as community-onset (75.4%) followed by hospital-ward acquired (13.4%), and ICU-acquired (11.2%). Urinary tract infections (42.2%), intra-abdominal (18.8%), lung (13.0%), vascular catheter (6.3%), and skin and soft tissue (5.2%) were the most common sources of infection with the majority of infections caused by monomicrobial gram-negative bacteria (71%). *E. coli* (43.8%), *Klebsiella* spp. (18.8%), and *Enterococcus* spp. (6.9%) were the most commonly isolated pathogens. Death at 90 days occurred in 261 patients (14.5%) in the 7-day group compared to 286 patients (16.1%) in the 14 days group (absolute difference −1.6%, 95% CI, −4.0 to 0.8). The median duration of antibiotics was 8 days vs 14 days in the 7-day and 14-day groups, respectively. The differences amongst the 7-day vs 14-day groups regarding ICU death was −0.6% (95% CI -3.2 to 1.9), in-hospital death −1.0% (95% CI -2.9 to 0.9), and relapse of bacteremia 0.4% (95% CI -0.6 to 1.4). Hospital length of stay, vasopressor use, mechanical ventilation, antibiotic-associated ADRs, and *C*. *difficile* infections were similar between groups. In the 7-day group, the median number of antibiotic-free days by day 28 was higher (19 days vs 14 days). The authors concluded that a clinician-driven 7-day treatment course was noninferior to a 14-day course among hospitalized bacteremic patients across a variety of infectious etiologies and pathogens.

Notable limitations of this study were the lack of power to detect differences in subgroup patient populations, and that the results do not apply to certain infectious disease syndromes or pathogens, such as fungal pathogens, *S. aureus*, and *S. lugdunensis*. In addition, severely immunocompromised hosts were not included.

### Dulhunty JM et al. Continuous vs Intermittent β-Lactam Antibiotic Infusions in Critically Ill Patients With Sepsis: The BLING III Randomized Clinical Trial

Sepsis is a severe and life-threatening response to infections that can lead to multi-organ failure requiring intensive care. In the treatment of infections causing sepsis, β-lactam antibiotics (such as piperacillin-tazobactam and meropenem) are commonly used.^54,55^ The effectiveness of these antibiotics relies heavily on maintaining optimal drug concentrations in the bloodstream over time which has led to different strategies for antibiotic administration (eg, extended and continuous infusions). However, the clinical benefits of continuous infusion of β-lactam antibiotics remain debated.^56^ The BLING III trial (β-Lactam Infusion in Sepsis) was a large, multi-center, international randomized controlled trial designed to evaluate the clinical outcomes of continuous vs intermittent β-lactam infusions in critically ill patients with sepsis.

The BLING III trial involved 7031 patients across 104 intensive care units across a variety of countries in Europe and Asia. Conducted from March 26, 2018, to April 12, 2023, the trial randomized adults with sepsis who had received piperacillin-tazobactam or meropenem within the prior 24 hours. Of those randomized, 7031 (97.6%) were included in the primary analysis. The most common infection sources were pulmonary (59.5%), intra-abdominal (13.0%), and bloodstream infections (8.0%). Nearly 70% of patients required vasopressors in the 24 hours prior to randomization, reflecting the acuity of illness. The median duration of treatment was 5.8 days (IQR 3.1-10.2) in the continuous group, and 5.7 days (IQR 3,1-10.3) in the intermittent infusion group. The most common sites of infection were pulmonary (59.5%) followed by intra-abdominal (13.0%) and blood (8.0%). Pathogenic organisms were identified in 40.8% of patients with Gram-negative bacteria being more common (68.8%) than Gram-positive (30.6%).

The primary outcome, 90-day mortality, in the continuous infusion group was 24.9% compared to 26.8% in the intermittent infusion group. However, this difference was not statistically significant (absolute difference −1.9%, 95% CI -4.9% to 1.1%; OR 0.91, 95% CI 0.81 to 1.01). Clinical cure rates at 14 days post-randomization were 55.7% vs 50.0% (OR 1.26, 95% CI 1.15 to 1.38), this outcome was statistically significant in favor of the continuous infusion group. Other secondary outcomes included all-cause ICU mortality, all-cause hospital mortality, and new acquisition, colonization, or infection with a multi-drug resistant organism (MDRO) showed no significant differences. The authors concluded that while continuous infusion β-Lactams showed trends toward better outcomes, the results did not provide strong enough evidence to recommend it over intermittent infusion in critically ill patients with sepsis.

Despite the numerical trends suggesting potential benefits with continuous infusion, these differences were not statistically significant, making it difficult to draw firm conclusions about the superiority of one method over the other. The study did not assess the impact of different pathogens on the outcomes of continuous vs intermittent infusions, which could have influenced the results. In addition, lack of minimum inhibitory concentration (MIC) data between groups further hinders adequate comparison as data suggests there can be correlation between dosing, MIC, and clinical outcomes. In real-world settings, adherence to continuous infusion protocols might be more challenging due to logistical issues. Finally, the trial focused on 90-day mortality and clinical cure, but it did not explore long-term outcomes like recurrence of infection, antibiotic resistance, or quality of life post-discharge, which could provide additional insights into the benefits or risks of continuous infusion.

### Gohil SK et al. Stewardship Prompts to Improve Antibiotic Selection for Pneumonia: The INSPIRE Randomized Clinical Trial

Pneumonia is the most common infection-related cause of hospitalization in adults and approximately half of these patients receive unnecessary broad-spectrum antibiotics.^57-60^ Efforts to reduce antibiotic duration or de-escalation do not focus on limiting unnecessary broad-spectrum antibiotic exposure prior to culture results.^61^ This trial was conducted to evaluate the impact of stewardship prompts integrated into the electronic health record (EHR), which were designed to guide clinicians towards guideline-recommended antibiotics for patients hospitalized with pneumonia.

The INSPIRE trial was a cluster-randomized clinical trial in 59 US community hospitals involving adults hospitalized with pneumonia within the HCA Healthcare system. The study consisted of 2 periods; an 18-month baseline period (April 2017-September 2018) and a 15-month intervention period (April 2019-June 2020). Hospitals were randomized to receive either the computerized provider order entry (CPOE) bundle (stewardship prompts) plus routine stewardship (eg, educational materials and quarterly coaching calls provided to providers) or routine stewardship (no prompts) alone. The intervention consisted of EHR-integrated CPOE prompts that appeared at the time of extended-spectrum antibiotic ordering and within the first 72 hours of admission, which recommended standard-spectrum antibiotics if the patient is non-ICU with MDRO risk <10%. MDRO risk was obtained from recursive partitioning models derived from retrospective data sets across 140 network hospitals which included factors such as health care exposures, antimicrobial exposure, and history or microbiologic evidence of MDROs. The primary outcome was extended-spectrum days of therapy (DOT) within the first 3 days of hospitalization (total number of different broad-spectrum antibiotics per each calendar day). Secondary outcomes were days of therapy of vancomycin and antipseudomonal agents. Safety outcomes were days to antibiotic escalation, days to ICU transfer, and length of stay.

Among 59 hospitals, there were 30 hospitals (47,029 patients) randomized to routine stewardship group and 29 hospitals (49,422 patients) randomized to the CPOE bundle intervention group. Extended-spectrum DOT for the CPOE bundle group decreased from 613.9 (baseline) to 428.5 (intervention) days. The overall rate ratio (RR) was 0.72 (95% CI 0.66 to 0.78, *P* < 0.001), indicating a 28.4% significantly decreased rate of primary outcome in the CPOE bundle group. Secondary outcomes of vancomycin DOT (RR 0.77, 95% CI 0.71 to 0.83, *P* < 0.001) and antipseudomonal DOT (RR 0.68, 95% CI 0.61 to 0.75, *P* < 0.001) were also reduced in the CPOE bundle group. Safety outcomes of percentage of ICU transfers and percentage of antibiotic escalation were similar between groups. Time to antibiotic escalation was 18.1% longer in the CPOE group (HR 0.82, 95% CI 0.69 to 0.97, *P* = 0.02). The CPOE bundle intervention resulted in a reduction in empiric extended-spectrum antibiotic use for pneumonia in non-critically ill adults.

Since the trial was conducted in community hospitals, applicability to other sites is undetermined. Culture results were also included regardless of specimen quality, potentially overestimating rates of MDROs. Additionally, the INSPIRE pneumonia prompts were conducted concurrently with urinary tract infection prompts, which could have contributed to alert fatigue. It also would be challenging to separate the effect of the prompts from those of education and feedback. Lastly, the newly updated pneumonia treatment guidelines could have impacted temporal trends during the study, but education of the guidelines was provided to both groups.

### Gohil SK et al. Stewardship Prompts to Improve antibiotic Selection for Urinary Tract Infection: The INSPIRE Randomized Clinical Trial

Urinary tract infections (UTIs) are one of the most common infections encountered in clinical practice, and improper antibiotic selection has been known to contribute to the rise of antibiotic resistance, particularly with MDROs. Broad-spectrum antibiotics (eg, fluoroquinolones) are often prescribed empirically for UTIs, increasing the risk of resistance and adverse patient outcomes. Antibiotic stewardship programs aim to optimize antibiotic use, but achieving this in real-world clinical settings can be challenging due to variability in clinician decision-making, particularly when treating infections without pathogen-specific data.^62-64^ Stewardship interventions, such as guideline-based recommendations or clinical decision support systems, can improve antibiotic prescribing.^63,64^ The INSPIRE UTI trial sought to evaluate the impact of such stewardship prompts on the selection of antibiotics for UTIs, aiming to reduce unnecessary use of broad-spectrum antibiotics without compromising patient safety or clinical outcomes.

The INSPIRE UTI trial was a cluster-randomized clinical conducted in parallel with the INSPIRE pneumonia trial using the similar methodology. The study consisted of 2 periods: an 18-month baseline period (April 2017-September 2018) and a 15-month intervention period (April 2019-June 2020). Hospitals were randomized to receive either the CPOE bundle (stewardship prompts) plus routine stewardship (eg, educational materials and monthly coaching calls provided to providers) or routine stewardship (no prompts) alone. The intervention consisted of EHR-integrated CPOE prompts that appeared at the time of extended-spectrum antibiotic ordering and within the first 72 hours of admission, which recommended standard-spectrum antibiotics if the patient had a low absolute risk (<10%) for MDRO UTI. MDRO risk was obtained from recursive partitioning models derived from retrospective data sets across 140 network hospitals which included factors such as health care exposures, antimicrobial exposure, and history or microbiologic evidence of MDROs. The primary outcome was extended-spectrum DOT within the first 3 days of hospitalization (total number of different broad-spectrum antibiotics per each calendar day). Secondary outcomes were days of therapy of vancomycin and antipseudomonal agents. Safety outcomes were days to antibiotic escalation, days to ICU transfer, and length of stay.

Among 59 hospitals, there were 30 hospitals (64,244 patients) randomized to routine stewardship and 29 hospitals (63,159 patients) randomized to the CPOE bundle intervention group. The implementation of CPOE prompts to guide antibiotic selection for UTIs led to a statistically significant 17.4% reduction in empiric use of extended-spectrum antibiotics from 41.1% to 33.9% (RR 0.83, 95% CI 0.77 to 0.89, *P* < 0.001). The rate ratio comparing days of therapy between groups for vancomycin (RR 0.89, 95% CI 0.82 to 0.96, *P* = 0.002) and antipseudomonal agents (RR 0.79, 95% CI 0.72 to 0.87, *P* < 0.001) were also significantly decreased in the intervention group. This was achieved without increasing ICU transfers, hospital length of stay, or *C. difficile* infections. Overall, the trial supports the integration of CPOE prompts into antibiotic stewardship programs to optimize UTI treatment and reduce unnecessary broad-spectrum antibiotic use.

The INSPIRE UTI trial has several limitations that should be considered when interpreting the results. First, the study’s cluster-randomized design, while robust, is observational in nature, which means causality cannot be definitively established. This introduces the possibility of unmeasured confounders that may have influenced the outcomes. Additionally, the trial was conducted within a single health system, limiting its generalizability to other healthcare settings with different patient populations, infrastructure, or resistance patterns. The findings may not apply to hospitals with lower resources or those in different geographic areas with different resistance profiles. Moreover, the trial focused on short-term safety outcomes, such as ICU transfers and *C. difficile* infections, but did not assess long-term impacts, such as antibiotic resistance trends or recurrent infections.

### Kaasch AJ et al. Efficacy and Safety of an Early Oral Switch in Low-Risk Staphylococcus Aureus Bloodstream Infection (SABATO): An International, Open-Label, Parallel-Group, Randomised, Controlled, Non-Inferiority Trial

*Staphylococcus aureus* bloodstream infections (SAB) are the leading cause of sepsis-related morbidity and mortality. Prolonged intravenous (IV) antibiotic therapy has historically been the mainstay due to the high risk of complications related to metastatic infections, infective endocarditis, and bacteremia persistence.^65,66^ While IV treatment has been the standard of care, risks associated with catheter-related bloodstream infections, thrombophlebitis, increased hospital length of stay, and higher healthcare costs can be associated with prolonged use.^67,68^ The possibility of transitioning to oral (PO) therapy earlier in treatment has gained special interest as a strategy to reduce those complications, but data is limited to confirm its safety and efficacy. The SABATO trial was designed to evaluate whether an early PO step-down therapy in low-risk SAB patients would be non-inferior to continued IV therapy as far as clinical outcomes.^19^

This open-label, multicenter, randomized controlled trial was conducted in 31 hospitals across Europe. Adult patients were enrolled if they had monomicrobial *S. aureus* bacteremia, received 5-7 days of effective IV therapy, and did not have evidence of infective endocarditis, persistent bacteremia, or a deep-seated infection. Eligible individuals were then randomized to either complete treatment with IV antibiotics or transition early to PO therapy. IV antimicrobials included standard of care anti-staphylococcal penicillins (eg, flucloxacillin, cloxacillin) or cefazolin for methicillin-susceptible *S. aureus* (MSSA) and vancomycin (with trough targets of 10-20 mcg/mL) or daptomycin 6-10 mg/kg for methicillin-resistant *S. aureus* (MRSA). Oral antibiotic regimens were selected by the study physician in the following order: sulfamethoxazole-trimethoprim (SXT) 800mg-160 mg twice daily for MSSA or MRSA, followed by clindamycin (CLI) 600 mg three times daily for MSSA or linezolid (LZD) 600 mg twice daily for MRSA.

A total of 213 patients were included in the intention-to-treat population, with 108 receiving oral therapy and 105 continuing IV therapy. Both groups received IV therapy for a median of 6 days (IQR PO 6-7 days, IV 5-7 days) and in full treatment course in for a median of 14 days (IQR 14-15 days). The primary composite outcome included all-cause mortality, bacteremia recurrence, or treatment failure within 90 days of randomization. The primary composite outcome occurred in 13% of patients in the oral switch group and 12% in the IV therapy group, with a difference of 0.7% (95% CI -7.8 to 9.1), meeting the non-inferiority criteria. There were no differences identified in any secondary endpoints (eg, survival at 14, 30, and 90 days, incidence of *C. difficile* infection, complications of IV administration) except a shorter length of stay in the oral switch group (12 vs 16 days, *P* = 0.043). Adverse events (eg, drug-related, new or recurrent infection, cardiac, respiratory, or constitutional symptoms) occurred in 34% of oral switch patients and 26% of IV therapy patients and were not statistically significantly different between groups. The authors concluded that oral step-down therapy could be an effective and safe alternative after careful assessment for certain low-risk SAB patients.

Despite its promising findings, the study had several limitations that are worth considering. Firstly, the strict inclusion criteria significantly reduced generalizability, as only 4.2% of screened patients were enrolled, limiting its applicability to how we commonly see the SAB population. The open-label design raises the possibility of bias, especially in subjective clinical assessments. Additionally, the adherence and compliance to oral therapy post-discharge was not strictly monitored, introducing a potential confounding factor that could’ve interfered with the study results. The study also lacked long-term follow-up beyond 90 days, making it unknown whether delayed recurrences or complications emerged later. Finally, variations in antibiotic selection for oral step-down therapy may have influenced outcomes, necessitating further research to determine optimal regimens that include but are not limited to dosing strategies and bioavailability of the oral medications.

### Karaba SM et al. Extended-Infusion β-Lactam Therapy, Mortality, and Subsequent Antibiotic Resistance Among Hospitalized Adults With Gram-Negative Bloodstream Infections

Gram-negative bloodstream infections (GN-BSIs) pose a significant challenge in healthcare due to their association with high morbidity and mortality, compounded by rising antimicrobial resistance. β-Lactam antibiotics remain a cornerstone of treatment, but optimizing their administration to improve outcomes and minimize resistance is an ongoing area of research. Traditionally, these antibiotics are given as intermittent infusions (Il-BL) over short periods, but extended-infusion β-lactam (EI-BL) therapy—administered over 3 or more hours—has been proposed to enhance efficacy, particularly against pathogens with elevated minimum inhibitory concentrations (MICs).^69,70^ Prior studies have yielded conflicting results on whether EI-BL improves survival or impacts resistance, prompting the need for further investigation.^71,72^ The study by Karaba et al, published in JAMA Network Open in July 2024, seeks to clarify these associations in a large cohort of hospitalized adults with GN-BSIs.

This was a retrospective cohort study analyzing 4861 adults with GN-BSIs across 24 U.S. hospitals in 2019. Patients were included if they received β-lactam antibiotics for at least 72 hours. The EI-BL group (352 patients) received infusions lasting 3 or more hours and the Il-BL group (4509 patients) received infusions of 1 hour or less. The primary outcome was 90-day mortality from blood culture collection, with secondary outcomes including recurrent infections, emergence of resistance, and adverse events. To balance baseline differences, propensity score matching (PSM) was applied in a 1:3 ratio, resulting in 1408 matched patients. Data were collected on demographics, comorbidities, illness severity (eg, Pitt bacteremia score), and microbiological details, with statistical analyses adjusting for confounders to assess EI-BL’s impact.

Among the matched cohort, 90-day mortality was lower in the EI-BL group (22%) compared to the II-BL group (28%), with an adjusted odds ratio (aOR) of 0.71 (95% CI, 0.52 to 0.97), suggesting a survival benefit. However, this advantage was confined to specific subgroups: patients with critical illness (aOR 0.47, 95% CI 0.28 to 0.81) and those with elevated β-lactam MICs (aOR 0.06, 95% CI 0.01 to 0.66). No significant difference was observed in recurrent infections (aOR 0.96, 95% CI 0.64 to 1.45) or resistance emergence (2.9% in EI-BL vs 7.2% in II-BL, *P* = 0.35). Adverse events, however, were more frequent with EI-BL, including catheter complications (aOR 3.14, 95% CI 1.66 to 5.96) and antibiotic discontinuation due to side effects like acute kidney injury (aOR 3.66, 95% CI 1.68 to 7.95).

The study’s retrospective design limits causal inference, and unmeasured confounders such as clinician decision-making may bias results. The small proportion of EI-BL patients (7.2%) and underpowered analysis of resistance (due to low recurrence rates) weaken generalizability and conclusions about resistance prevention. Variability in hospital practices and lack of data on infusion timing relative to infection onset further complicate interpretation. Despite these limitations, the findings suggest EI-BL therapy reduces mortality in severely ill patients or those with less susceptible pathogens, though benefits must be weighed against increased adverse events. The authors call for larger, prospective studies to confirm these results and explore resistance implications, reinforcing EI-BL’s potential role in targeted GN-BSI management.

### López-Cortés LE et al. Efficacy and Safety of a Structured De-escalation From Antipseudomonal β-Lactams in Bloodstream Infections Due to Enterobacterales (SIMPLIFY): An Open-Label, Multicentre, Randomised Trial

Broad-spectrum β-lactams are often overprescribed in the setting of bloodstream infections (BSI) due to concern for inadequate empiric coverage. Although some of these agents provide reliable activity against *P. aeruginosa* and other resistant pathogens, their prolonged use has been associated with significant consequences, including disruption of the gut microbiome that can lead to development of *C*. *difficile* infection as well as selection for MDROs.^73,74^ Antimicrobial stewardship programs emphasize de-escalation to narrower spectrum agents or even at the empiric phase if a patient doesn’t meet certain risk factors, but data supporting this practice in BSI due to Enterobacterales remain limited.^75,76^ The SIMPLIFY trial aimed to evaluate the clinical outcomes to determine if a structured de-escalation approach from antipseudomonal β-lactams to narrower-spectrum β-lactams in Enterobacterales BSI was non-inferior to continued broad-spectrum therapy.^77^

This was an open-label, multicenter, randomized controlled trial conducted across different hospitals in Spain. Screened adult patients with BSI due to Enterobacterales were included in the study if they had received at least 48 hours of effective antipseudomonal β-lactam therapy, met criteria for being hemodynamically stable, and had a documented narrower β-lactams that was sensitive to the pathogen from the blood culture. Eligible participants were randomized to either continue antipseudomonal therapy or de-escalate to a narrower-spectrum β-lactam, such as ceftriaxone or amoxicillin-clavulanate. Randomization was stratified by study site, and the primary outcome of clinical cure was assessed 3-5 days after completion of antibiotic treatment including resolution of all symptoms and signs of infection and no need for treatment modification due to an unfavorable clinical response or adverse effect.

The final analysis included 331 patients, 164 in the de-escalated therapy group and 167 in the broad-spectrum group. The baseline characteristics for both groups were roughly similar with predominantly biliary tract and urinary tract infections due to *E. coli* and *K. pneumoniae*. However numerical differences were noted as more patients in the de-escalation group had nosocomial infections and used immunosuppressive medication, and fewer patients in the de-escalation group presented with sepsis. The primary outcome of clinical cure was achieved by 90% and 89% of patients in the de-escalation group compared to the control group (risk difference 1.6%, 95% CI -5.0 to 8.2) There were no differences in secondary endpoints such as microbiological cure, clinical cure at 60 days, recurrence until 60 days, 60-day mortality, and *C. difficile* infection until day 60. These findings suggest that structured de-escalation in stable Enterobacterales BSI patients is a safe and effective strategy that may reduce antimicrobial resistance pressure without sacrificing the priority of clinical success.

Several limitations were acknowledged by the authors. The open-label design introduced the potential for bias in a subjective clinical endpoint such as determination of clinical cure. Additionally, these findings are only applicable to patients with confirmed susceptibility to narrower-spectrum agents, which limits its application in the cases where susceptibility results were either delayed or unavailable. Since the trial was conducted in hospitals with the support of an antimicrobial stewardship programs, generalizing to institutions with limited support and resources could be difficult. Another limitation was the lack of longer-term follow-up to assess the impact of de-escalation on not only antimicrobial resistance trends but development of C. difficile. Furthermore, variations in geographical resistance patterns and local antibiograms may influence de-escalation patterns, making external validation a key prior to widespread adoption.

### Ranganath N et al. Short vs Prolonged Duration of Therapy for Pseudomonas Aeruginosa Bacteraemia: A Systematic Review and Meta-Analysis

*Pseudomonas aeruginosa* bacteremia is a severe infection primarily affecting immunocompromised and hospitalized patients. Growing concerns about antibiotic resistance, toxicity, and healthcare costs have piqued interest in shorter treatment durations. The optimal duration for *P. aeruginosa* bacteremia remains unsettled due to limited high-quality data. This prompted Ranganath et al.’s systematic review and meta-analysis, published in the Journal of Hospital Infection in 2024, to review existing evidence and guide clinical practice in an era prioritizing antimicrobial stewardship.

Ranganath, et al conducted a systematic review following PRISMA guidelines, searching PubMed, Embase, and Cochrane Library databases for studies up to December 2023 comparing short-course (≤10 days) vs prolonged-course (>10 days) antibiotic therapy for P. aeruginosa bacteremia. Eligible studies included randomized controlled trials (RCTs) and observational studies involving adults with confirmed bacteremia, excluding polymicrobial infections. Outcomes assessed were 30-day mortality, relapse (recurrent bacteremia or infection), and adverse events (eg, *C*. *difficile* infection). Of the 908 identified studies, six were included in the systematic review and five studies with head-to-head comparison of treatment duration were assessed in the meta-analysis, totaling 1746 patients. Data was pooled using random-effects meta-analysis, with heterogeneity assessed via I^2^ statistics and bias evaluated using the ROBINS-I tool for observational studies and Cochrane Risk of Bias for RCTs.

Across the pooled cohort, short-course therapy (median 7 days) showed no significant difference in 30-day mortality compared to prolonged-course therapy (median 14 days), with a risk ratio of 0.92 (95% CI 0.74 to 1.15; I^2^ = 34%). Relapse rates were similarly comparable (risk ratio 1.12, 95% CI 0.68 to 1.84; I^2^ = 19%), with 4.1% (22/534) of short-course patients and 3.6% (20/553) of prolonged-course patients experiencing recurrence. Subgroup analyses by study design, illness severity, or source control (eg, catheter removal) revealed no notable differences. Adverse events were less frequent with short-course therapy (risk ratio 0.61, 95% CI 0.39 to 0.95), particularly for *C. difficile* infections and antibiotic-related toxicity. Overall, the evidence suggests noninferiority of shorter durations, though heterogeneity and study quality varied.

The analysis was constrained by the number of observational studies which can introduce potential confounding. There was variability in definitions of “short” and “prolonged” durations, as well as antibiotic regimens and source control practices across the studies which may have influenced outcomes. The exclusion of polymicrobial infections also limited generalizability. Despite these limitations, the findings support short-course therapy (7-10 days) as a viable option for *P. aeruginosa* bacteremia. Short course therapy showed similar efficacy to prolonged courses with fewer adverse events. The authors advocate for larger RCTs to confirm these results, emphasizing that clinical judgment—considering patient stability and source control—remains critical in tailoring therapy duration.

### Tamma PD et al. Infectious Diseases Society of America 2024 Guidance on the Treatment of Antimicrobial-Resistant Gram-Negative Infections

The 2024 iteration of this guidance document provides updates on management of infections caused by drug-resistant bacteria focusing specifically on extended-spectrum-β-lactamase-producing Enterobacterales (ESBL-E), AmpC β-lactamase-producing Enterobacterales (AmpC-E), carbapenem-resistant Enterobacterales (CRE), *P*. *aeruginosa* with difficult-to-treat resistance (DTR *P. aeruginosa*), carbapenem-resistant *Acinetobacter baumanii* (CRAB), and *Stenotrophomonas maltophilia*. The main updates are summarized below.

ESBL-E:

• Cystitis
  • Although amoxicillin-clavulanic acid is not a preferred agent for uncomplicated ESBL-E cystitis, if it is prescribed because resistance or toxicities preclude use of alternative oral antibiotics and there is a preference to avoid IV antibiotics, caution should be given to patients about the potential increased risk of recurrent infection if used.
• Pyelonephritis/cUTI
  • Fosfomycin is not suggested for the treatment of pyelonephritis or cUTI. In the U.S., IV fosfomycin is not clinically available however transitioning to daily oral fosfomycin needs further investigation though it may be reasonable option when preferred or alternative oral options are not available.
  • Based on updated and re-reviewed clinical data showing similar cure rates with ceftolozane/tazobactam compared to meropenem, ceftolozane/tazobactam was added as a β-lactam/β-inhibitor option for the treatment of infections caused by ESBL-E, however it should be preserved for use against DTR *P. aeruginosa* when possible.^78-80^

AmpC-E:

• Clarification was provided that even without upregulation of AmpC production, basal production of AmpC β-lactamases in organisms such as *Enterobacter cloacae, Klebsiella aerogenes*, or *Citrobacter freundii*, leads to intrinsic resistance to ampicillin, amoxicillin-clavulanic acid, ampicillin-sulbactam, and first- and second-generation cephalosporins.
• The dosing of cefepime 2 grams every 8 hours with an extended infusion over 3 hours was added for isolates with a cefepime susceptible dose-dependent (SDD) MIC 4-8 mcg/mL.

CRE:

• The dosing of ceftazidime-avibactam and aztreonam was updated as every 8-hour infusions to facilitate simultaneous administration.

DTR *P. aeruginosa*:

• High-dose, extended-infusion traditional β-lactams (eg, cefepime, piperacillin-tazobactam) are recommended if susceptible, even when carbapenems test non-susceptible.
• U.S. surveillance data is summarized for the new β-lactams (ceftolozane-tazobactam 90%, ceftazidime-avibactam 85%, imipenem-cilastatin-relebactam 86%, cefiderocol 99%), and an emphasis on regional differences should be noted to guide empiric treatment (eg, MBL-production in Latin America and the Middle East, GES-production in Spain, KPC-production in Latin America and China).^81-84^
• Once daily tobramycin or amikacin was added as an alternative for pyelonephritis or cUTI given the favorable pharmacokinetics of aminoglycosides for these indications.

CRAB:

• Sulbactam-durlobactam in combination with meropenem or imipenem-cilastatin was added as a preferred agent for infections due to CRAB based on the ATTACK trial.^85^ While the clinical benefit of combination therapy is still unclear, several in vitro studies note a potential 1-to 2-fold lowering of sulbactam-durlobactam MICs and enhanced bactericidal activity likely due to PBP saturation from sulbactam and imipenem.^86,87^
• High-dose ampicillin-sulbactam (9 grams every 8 hours infused over 4 hours or 27 grams continuous infusion) as a component of combination therapy is an alternative when sulbactam-durlobactam is unavailable.

Stenotrophomonas maltophilia:

• The minimal robust data of combined studies suggest poor pharmacokinetic/pharmacodynamic (PK/PD) properties of individual agents.
• Combination therapy with two agents is listed in order of preference: cefiderocol (with a second agent at least initially), ceftazidime-avibactam and aztreonam, minocycline, trimethoprim-sulfamethoxazole, and levofloxacin.
• Tigecycline is no longer recommended as a component of combination therapy due more favorable PK/PD and side effect profile with minocycline.

### Tingsgård S et al. Early Switch From Intravenous to Oral Antibiotics for Patients With Uncomplicated Gram-Negative Bacteremia

The increasing incidence of gram-negative bacteremia, including cases with antibiotic-resistant organisms, is associated with significant morbidity and mortality.^88,89^ Management of gram-negative bacteremia traditionally involves prolonged IV antibiotic therapy, necessitating extended hospital stays. Fueled by the desire to reduce hospital length of stay, health care costs, and improve patient quality of life, recent studies have examined optimal treatment duration for gram-negative bacteremia and the transition from IV to oral antibiotic therapy.^90-92^ However, there has been a lack of emphasis on optimal timing for de-escalation from IV to oral antibiotic. In this study, Tingsgård S et al investigated the safety and efficacy of an early switch to oral antibiotics in patients with uncomplicated gram-negative bacteremia.

The study employed a cohort design, using a target trial emulation to include observational data from patients in 4 Denmark hospitals from January 2018 to December 2021. Adults with gram-negative bacteremia, who were clinically stable and had available susceptibility report within 4 days of initial blood culture, were assigned to 1 of 2 treatment arms. One arm was transitioned early to oral antibiotics within 4 days of blood culture, the other arm was continued on IV antibiotics for at least 5 days. The choice of oral antibiotic was guided by susceptibility testing and physician discretion. Primary outcome was 90-day all-cause mortality. Of 914 eligible patients, 433 (47.4%) were assigned to early oral antibiotics transition arm and 481 (52.6%) were assigned to receive prolonged IV antibiotics. Patients who received prolonged IV therapy were older (76 vs 73 years), had more comorbidities (median Charlson Comorbidity Index of 5 vs 4), and had more complicated progression of the bacteremia. Patients who were transitioned early to oral antibiotics were more likely to have community-acquired bacteremia (89.4% vs 80.9%), with urinary tract as the source (83.1% vs 70.5%). Oral β-lactams were most commonly prescribed (63.1%) followed by oral ciprofloxacin (16.6%). During follow-up, the proportion of patients who died was higher in the prolonged IV antibiotics group compared to the early oral transition group (14.3% vs 6.9%). Ninety-day all-cause mortality, in the intention-to-treat analysis, was 9.1% for the early switch arm and 11.7% for the group with prolonged IV therapy (risk difference (RD) −2.5%, 95% CI -5.7 to 0.7). In the per-protocol analysis, 90-day all-cause mortality was 9.6% for the early switch group and 9.7% for the other group (RD -0.1%, 95% CI -3.4% to 3.1%). These results indicated comparable rates of 90-day all-cause mortality between two groups.

Despite the promising results, this study is not without limitations. As an observational study, important confounders may have been missing or incompletely documented. Additionally, incidence of MDROs was low, potentially impacting the applicability of this study. Lastly, patients with complicated gram-negative bacteremia were excluded from the analysis, limiting the applicability of the results to uncomplicated cases with rapid improvement. Nonetheless, this study contributes valuable insights into the potential for optimizing antibiotic therapy in gram-negative bacteremia, emphasizing the importance of individualized treatment approaches and the potential for safely reducing the duration of IV antibiotic use.

## Conclusion

Advances in ID research included an emphasis of shorter vs longer durations of treatment for invasive infections, antibiotic de-escalation strategies, oral step-down therapies, and the effects of extended infusion beta-lactams. This review provides a summary of the most influential ID literature recently published as voted by ID practitioners in the HIDN and SIDP network with aims to update clinicians and other relevant stakeholders on the current potential practice changing ID pharmacotherapy publications from 2024.
